# Contrast-enhanced multispectral optoacoustic tomography for the assessment of the gastrointestinal transit in patients with cystic fibrosis

**DOI:** 10.1016/j.pacs.2025.100766

**Published:** 2025-09-10

**Authors:** Johanna Fuchte, Felix Wachter, Merle Claßen, Hannah Vogt-Wolz, Lars-Philip Paulus, Henriette Mandelbaum, Adrian Buehler, Gregor Siebenlist, Jörg Jüngert, Joachim Wölfle, André Hörning, Ferdinand Knieling, Adrian P. Regensburger, Alexander Schnell

**Affiliations:** Department of Pediatrics and Adolescent Medicine, University Hospital Erlangen, Germany

**Keywords:** Optoacoustics, Photoacoustics, Multispectral optoacoustic tomography, Intestinal transit, Indocyanine green, Cystic fibrosis

## Abstract

Cystic fibrosis (CF) affects the gastrointestinal tract, but assessing gastrointestinal transit usually requires invasive procedures or exposure to ionizing radiation. Contrast-enhanced multispectral optoacoustic tomography (CE-MSOT) offers a novel, non-invasive, and radiation-free approach to assess gastrointestinal function by orally administered dyes. In this clinical pilot-study five patients with cystic fibrosis and four healthy volunteers received CE-MSOT before and 6-times hourly after a standardized breakfast with Indocyanin green (ICG) as dye. The gastric antrum, terminal ileum and sigmoid colon were recorded and MSOT signals spectrally unmixed to detect ICG signals to determine the transit time. ICG excretion was confirmed by fluorescence imaging of stool samples. MSOT ICG signals were detected earlier in the terminal ileum of CF patients, reaching a maximum after 120 min (p = 0.0079), compared to 240 min (p = 0.0286) in healthy controls after ICG intake (p = 0.0159). In CF patients, ICG signal was further detected in the sigmoid colon from 240 min onwards (p = 0.0079 after 300 min). But, no significant changes in the ICG signal were observed in the sigmoid colon of controls. Furthermore, signals of ICG were verified in 12 of 19 stool samples by fluorescence imaging. In this study, we demonstrated the potential of CE-MSOT for functional imaging of the intestine in CF patients and revealed faster intestinal transit in CF patients compared to healthy controls.

## 1 Introduction

Cystic fibrosis (CF) is the most common hereditary disease in Central Europe, with an incidence of around 3300 to 4800 new cases [1]. The disease follows an autosomal recessive inheritance pattern and is caused by a mutation in the cystic fibrosis transmembrane conductance regulator (CFTR) gene, which regulates the Cl^−^ channel [2]. Beside many other organ systems, the gastrointestinal tract can also be affected [3]. Gastrointestinal manifestations, including reduced appetite, flatulence, bloating, abdominal pain, and steatorrhoea, significantly impact the quality of life of pwCF [4], [5]. A CF-specific gastrointestinal manifestation is the distal intestinal obstructive syndrome [6]. It is characterised by accumulation of fecal mass in ileocoecum resulting in unspecific symptoms as abdominal pain, nausea, vomiting and a distended abdomen. The annual reports of the cystic fibrosis registry show that even the introduction of CFTR modulators did not lead to a significant reduction in incidence. So, early diagnosis is still critical and affects children and adults equally [7]. Diagnosis is possible based on abdominal radiographs but in order to economise radiation for children, alternative methods are warranted [8], [9].

Optoacoustic imaging techniques offer new diagnostic options in various diseases and clinical applications [10], [11], [12] especially in cancer [13], [14], [15], inflammation [16] and cardiovascular diseases [17]. In MSOT, similar to conventional sonography, a transducer is placed on the skin and instead of sound, energy is applied to the tissue by means of flashes of light. The optical energy is absorbed by endogenouse tissue chromophores such as haemoglobins or exogenouse chromophores (dyes). This results in localized heating and expansion generating detectable acoustic pressure waves [18], [19], [20]. Besides the potential of MSOT to non-invasively measure hemoglobin signals as a surrogate marker for inflammatory activity of the intestine [21], [22], previous studies have shown that the orally ingested dye indocyanine green (ICG) can be used to asses the gastrointestinal transit in healthy human subjects [23] or with nanoparticles in preclinical scenarios [24].

Our study aims to investigate the feasibility and clinical utility of contrast-enhanced MSOT (CE-MSOT) for non-invasive assessment of gastrointestinal transit in CF patients, providing valuable insights into the pathophysiology of gastrointestinal dysfunction in this population.

## 2 Methods

### 2.1 Design and flow of the study

Between October 2023 and August 2024, patients from the Cystic Fibrosis Center of the Department of Pediatrics and Adolescent Medicine of the Friedrich-Alexander-University Erlangen-Nürnberg and healthy controls were examined in this study. The study was approved by the local ethics committee (voting number 23–58-B), registered (NCT06063785) and conducted in accordance with the Declaration of Helsinki. All study participants provided written informed consent.

General exclusion criteria were pregnancy, breastfeeding mothers, tattoos in the area of the examination and subcutaneous fat tissue over 3 cm. With regard to ICG, known hypersensitivity to ICG, sodium iodide or iodine, hyperthyroidism, focal or diffuse thyroid autonomy or a treatment close in time to check thyroid function with ingestion of radioactive iodine within two weeks before or after the study were exclusion criteria. Impaired kidney function or the use of the following medications also led to exclusion: Beta-blockers, anticonvulsants, cyclopropane, bisulphite compounds, haloperidol, heroin, meperidine, metamizole, methadone, morphine, nitrofurantoin, opium alkaloids, phenobarbital, phenylbutazone, probenecid, rifampicin, any injection containing sodium bisulphite. Exclusion criteria for the cohort were the use of systemic glucocorticoids or immunosuppressants as part of long-term medication or an acute exacerbation of infection. For the healthy control patients, the presence of liver disease and the use of systemic glucocorticoids or immunosuppressants as part of long-term medication were considered exclusion criteria.

**Fig. 1:**
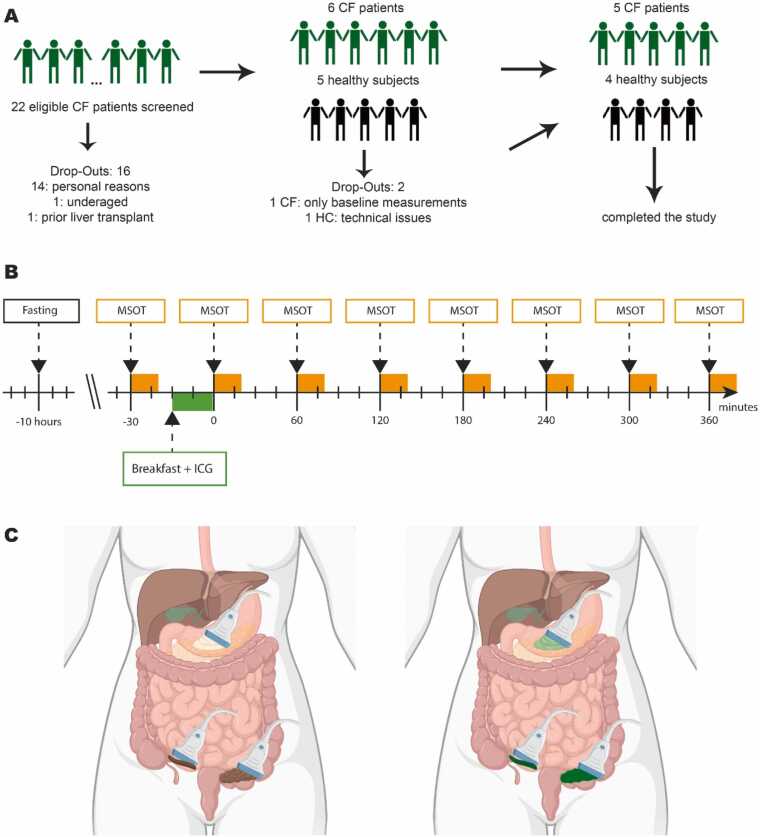
Study flow. A: 22 eligible patients of the Cystic Fibrosis Center of the Department of Pediatrics and Adolescent Medicine of the Friedrich-Alexander-University Erlangen-Nuremberg were screened. In addition to 6 selected pwCF, 5 healthy control probands also participated in the study. One participant of the group of pwCF and one healthy control proband were excluded while 5 pwCF and 4 healthy subjects completed the study. B: All subjects received a breakfast with ICG after fasting from the previous evening on. MSOT was performed directly before, after and 6 times hourly after ICG intake. Image were created with Illustrator. C: Three intestinal segments (gastric antrum, terminal ileum and sigmoid colon) were assessed. MSOT uses pulsed laser light to generate thermoelastic expansion. The optoacoustic absorption spectra can be used to spectrally unmix for endogenous (hemoglobin, HB) and exogenouse (ICG) chromophores in the intestinal segments. Created with biorender.com.

Before the day of the examination, all subjects fasted from 10 p.m. the previous day until the start of the examination at 8 a.m. All study participants underwent an ultrasound examination of the liver (including acoustic radiation force impulse imaging, ARFI, data not included in this manuscript) and hybrid MSOT/Reflectance ultrasound computed tomography of the intestine. Subsequently, all participants received a standardized breakfast (100 g fat-containing yoghurt and 50 g muesli as well as 50 mg ICG). Immediately after ingestion, a further measurement was taken, followed by six additional intestinal measurements using MSOT, each 60 min apart (Fig. 1). Anatomical locations for imaging were as follows: gastric antrum, terminal ileum and sigmoid colon. Furthermore, all participants were given three stool tubes to take home for the first three stool excretions after the examination.

### 2.2 MSOT device

Multi-spectral optoacoustic tomography (MSOT) was performed using an Acuity ECHO CE system (iThera Medical GmbH, Munich, Germany) to visualize intestinal segments. This system integrates ultrasound (B-mode) with optoacoustic imaging to enhance anatomical localization [25]. A handheld 2D detector, combined with transparent ultrasound gel (Aquasonic Clear, MDSS GmbH, Hannover, Germany), was used to capture a minimum of three images per location. During imaging, both subjects and all treating persons wore laser safety goggles. Anatomical regions were identified using B-mode imaging, while optoacoustic signals were recorded at wavelengths of 700, 730, 760, 800, 850 and 900 nm.

The analysis was performed using the iLabs software (V 1.3.25, iThera Medical GmbH, Munich, Germany) provided by the manufacturer. Regions of interest (ROIs) were defined within the intestinal lumen directly after image acquisition using B-mode guidance and verified by a second reader. The presence of stool in the bowel lumen served as an anatomical landmark. Following data transfer via the iLabs software, a second reader reviewed and adjusted the ROIs as needed to ensure consistency across scans. To quantify individual wavelengths and unmixed ICG signal levels (derived from wavelengths 700, 730, 760, 800 and 850 nm), the mean of the highest 10 % of pixels within each ROI was used in batch mode analyses. Spectral unmixing for ICG was performed using a 6.5 µM plasma reference spectrum, based on prior studies [23].

### 2.3 CFabd-score

**Fig. 2:**
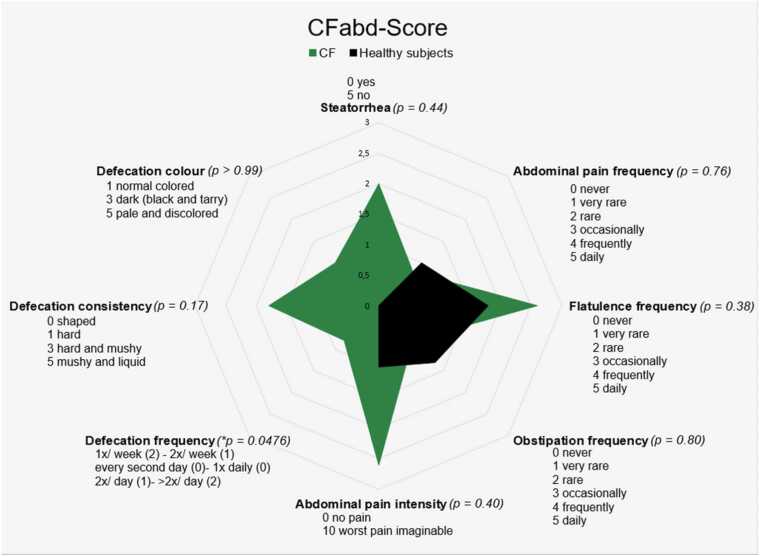
Modified CFabd-Score. Healthy subjects (black) and pwCF (green) means of modified CFAbd score.

As abdominal involvement in Cystic Fibrosis is not fully understood, a questionnaire was designed to identify an appropriate method for quantifying gastrointestinal symptoms [26], [27]. Our aim was to assess common abdominal symptoms in pwCF in a meaningful and practical manner using a modified version of the CFabd Score. The items included the frequency and intensity of abdominal pain, the frequency of flatulence, the frequency of obstipation, steatorrhea, the frequency and consistency of defecation and the color of stool. Participants were asked to characterize their symptoms on a scale, with each point clearly defined (Fig. 2).

### 2.4 Fluorescence imaging

After the examination, 19 stool samples from 4 pwCF and 3 controls were collected and examined for the presence of ICG using fluorescence imaging as previously described [23]. An in vivo imaging system (IVIS Spectrum, PerlkinElmer Inc., Waltham, MA, US) was used to examine the fluorescent properties of these stool samples. Excitation wavelengths of 570, 605, 640, 675, 710 and 745 nm and emission wavelengths of 760, 780, 800, 820 and 840 nm were used. For the spectral unmixing of the ICG signals in the stool samples, the factory presets for ICG fluorescence and tissue autofluorescence signals were used.

### 2.5 Further medical examinations

PwCF received a fasting blood sample. In addition to a small blood count and coagulation parameters, liver and bile values were also examined (data not shown). Furthermore, the current FEV1 and pseudomonas status were documented.

### 2.6 Statistics

Statistical analysis was performed using GraphPad Prism software (Version 10.2, GraphPad Software Inc., San Diego, USA). The demographic data of all subjects were expressed as numbers and percentages or as mean and standard deviation. We did not assume a normal distribution. Absolute ICG values and normalized ICG values to the initial value at time point 0 (Normalized Value in % = absolute Time-point value/ absolute Baseline value * 100) were compared between HC and pwCF. Due to technical reasons, we did not obtain a measured value for one of the subjects (pwCF) timepoint 0 (subject 4). Therefore, we used the lowest measured value of another participant from the cohort of pwCF at this time for normalization purposes. We performed mixed effect analysis to examine differences between groups for each dependent variable at each phase. After that the ICG signals were tested at different time points using Mann-Whitney tests. P-values < 0.05 indicate statistical significance.

## 3 Results

### 3.1 Clinical trial

From October 2023 to August 2024, a total of 22 pwCF were screened for study inclusion at the Department of Child- and Adolescent Medicine at the University Hospital Erlangen. N = 6 pwCF and n = 5 healthy volunteers were enrolled, all aged over 18 years. N = 5 pwCF and n = 4 controls successfully completed the study protocol (Fig. 1 and Supplementary Table 1). Baseline characteristics of the study cohorts are summarized in Table 1.

**Table 1:** Demographic data.

	HC	CF
Patients (n)	4	5
Age (years)	25.0 ± 2.7	29.2 ± 7.7
Height (kg)	177.5 ± 7.9	170.4 ± 11.0
Weight (kg)	69.0 ± 5.3	69.4 ± 12.8
BMI (kg/m^2)	22.0 ± 2.3	23.7 ± 1.8
FEV1 (%)	/	96.2 ± 16.1
Pseudomonasstatus	/	0 (0 %)
Sex F	2 (50 %)	1 (20 %)
Mutation		
F508del/F508del	/	5 (100 %)
Pretreatment with CFTR modulator other than ETI	/	
IVA	/	5 (100 %)
UDCA medication (20 mg/kg BW)	0 (0 %)	3 (60 %)

Data in Table 1 characterize the cohorts. Values are numbers and percentage or mean ± standard deviation (SD).

### 3.2 Modified CFabd score

Both, pwCF and healthy controls filled in a modified version of the CFabd score to quantify their gastrointestinal symptoms. We could detect a higher score in almost every category in the pwCF, however only statistically significant in the defecation frequency (p = 0.0476) (Fig. 2 and Supplementary Table 2).

### 3.3 Contrast-enhanced MSOT in pwCF and control subjects

**Fig. 3:**
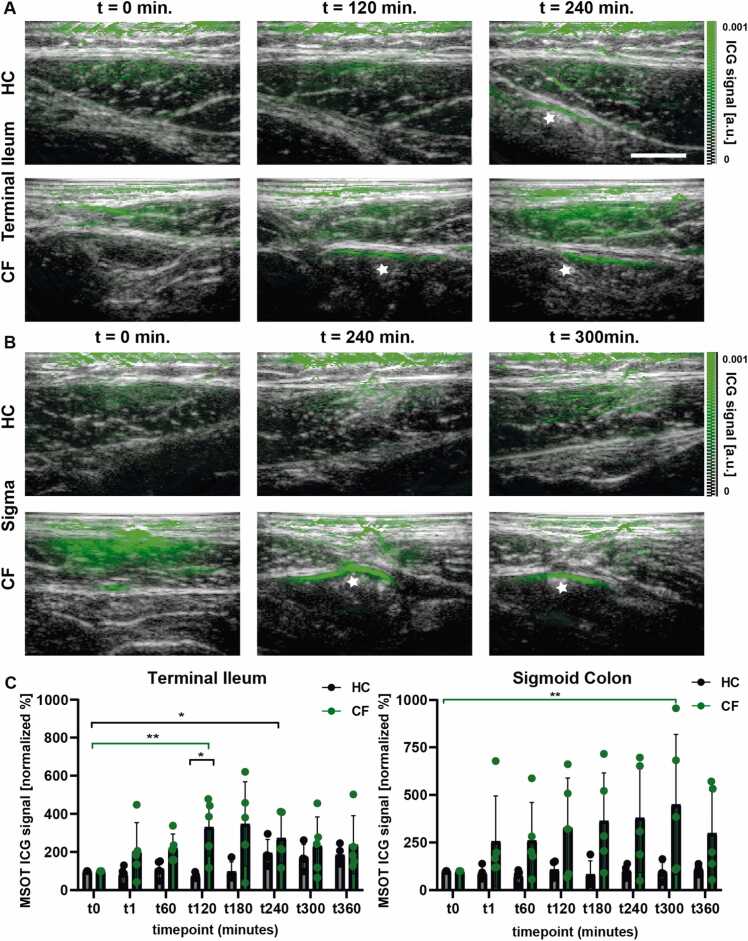
Contrast-enhanced MSOT for the assessment of gastrointestinal transit. A: Image of ICG signals in healthy control subjects and in pwCF in the terminal ileum before ICG intake, after 120 min and after 240 min. Stoolshowing ICG-signals is marked by white stars. White bar represents 1 cm. ICG = Indocyanin Green, HC = Healthy Controls, CF = Cystic Fibrosis. B: MSOT ICG signals were detected 240 and 300 min after oral ICG intake in pwCF in the sigmoid colon. In contrast, no ICG signals were detected in healthy control subjects. Stoolshowing ICG-signals is marked by white stars. C: Quantification of the MSOT-ICG signals in the terminal ileum and sigmoid colon, one in healthy subjects (black) and one in pwCF (green). Dots represent single values of each study participant, bars and whiskers represent mean ± SD. Asterisks mark statistically significant increases (* *p* < 0.05, ** *p* < 0.01).

At the beginning of our study, no relevant ICG signals were detected throughout all observed intestinal segments. For the gastric antrum, we could not detect any relevant changes of the ICG signal (see Supplementary Figure 1). However, for terminal ileum, pwCF demonstrated rapid signal increases which reached its maximum 120 min after oral ICG intake compared to the baseline values (330.8 % vs. 100 %, p = 0.0079). Additionally, the measured values of both cohorts differed significantly at this timepoint (330.8 % vs. 74.8 %, p = 0.0159). After this maximum, we noticed a relatively rapid decline in the signal. In contrast, healthy controls showed an slower increase and reached its maximum after 240 min (194.3 % vs. 100 %, p = 0.0286). During the remaining time of the investigation, the signal in the terminal ileum remained relatively constant (Fig. 3). In cases of OAI ICG detection, a specific OAI peak at 800 nm could be detected (Supplementary Figure 2). In contrast, the spectrum of stool differs significantly from that of ICG, not showing a signal peak at 800 nm (Supplementary Figure 3). In addition, each individual signal history is presented in Supplementary Figure 4.

We also tested for general differences of the ICG signal in the terminal ileum in the longitudinal course of the study by using a 2-way-ANOVA. Here, we found a statistically significant difference between the two cohorts (p = 0.0479) for the entire observation period. Absolute values for the terminal ileum were also tested, but no significant differences were detected (see Supplementary Figure 5).

In the sigmoid colon, there was no significant signal increase in the control group during our study period. Interestingly, 3 out of 5 pwCF showed an increase and a significantly increased signal could be detected after 240 min. The detected signal lasted until the investigation was completed (100 % vs. 449.2 % after 300 min, p = 0.0079) (Fig. 3). Again, each individual signal history is presented in Supplementary Figure 6.

Absolute values for the sigmoid colon were also tested, but no significant differences were detected (see Supplementary Figure 7).

### 3.4 Proof of successful gastrointestinal passage

**Fig. 4:**
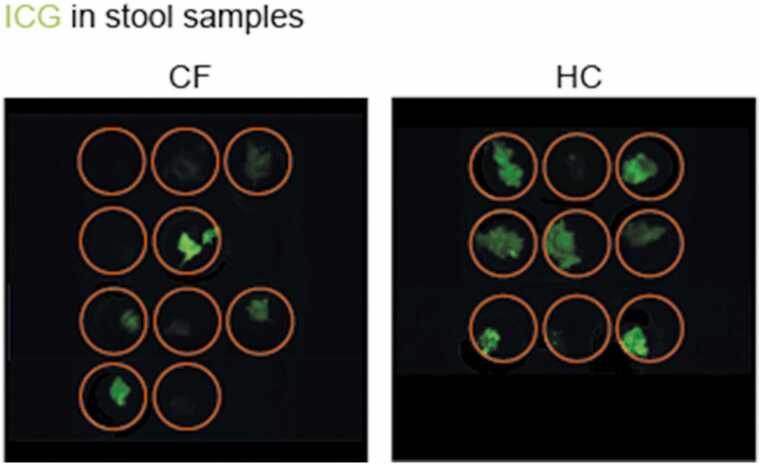
ICG stool samples: ICG excretion in the stool was confirmed by fluorescence imaging in both pwCF (left) and healthy control subjects (right). Each row represents one study participant. Subjects were asked to collect three consecutive stool samples after the examination (2–3 circles in each row).

Two to three stool samples were collected from participants to confirm ICG passage through the gastrointestinal tract without systemic uptake or intestinal degradation. Fluorescent imaging was performed on 19 stool samples from n = 4 pwCF and n = 3 subjects. The information on the sequence of the samples was not reliably provided by the participants. Nevertheless, we could identify ICG in 12 of the 19 stool samples, both in pwCF and controls, confirming successful excretion (Fig. 4).

## 4 Discussion

In this study we demonstrated the ability of CE-MSOT to asses the gastrointestinal transit in pwCF. Compared to healthy subjects, pwCF exhibited faster intestinal transit. In the first instance, this finding seems to be contradictory to the fact that gastrointestinal transit time has been reported to be prolonged in pwCF in comparison to healthy individuals [28], [29]. Accordingly, an study relying on MRI measurements revealed a longer oro-cecal transit time in pwCF (330 min) compared to healthy controls (210 min) [30]. Importantly, while our study observed significantly different transit times in pwCF, the transit times in healthy subjects in our study match those of the MRI measurements which reflects the general reliability of the MSOT method.

This finding, which stands in contrast to the previous reported intestinal transit times in pwCF, might be attributed to the influence of highly effective CFTR modulator therapy Elexacaftor – Tezacaftor – Ivacaftor, which was introduced to the European market in August 2020. Hence, as the previously cited MRI study was submitted in spring 2020, their reported findings reflect the gastrointestinal situation of pwCF in the pre-ETI era. In contrast to that, all of our recruited patients did receive the highly effective treatment with ETI.

Although, the precise effect of modulator therapy on gastrointestinal symptoms remains an area of active investigation [31], it is well-established that CFTR modulator therapies have revolutionized CF care by improving lung function, sweat chloride concentration, and body mass index [32] as a result of direct functional CFTR restoration. Interestingly, a recently published study by Yule et al. indicated a shortened transit time in pwCF as a result of ETI [33]. Therefore - given the recognized interplay between CFTR function, gut microbiota composition and gastrointestinal motility and the fact that all the pwCF participating in our study did receive this new therapy which directly restores CFTR function - we attribute our findings of a faster intestinal transit time directly the CFTR modulator treatment [34].

Furthermore, we detected the ICG signal in the sigmoid colon of pwCF during the investigation, while it was not visible in the control group. A previous study demonstrated a detectable ICG signal in the sigmoid colon of healthy subjects 24 h after ICG intake [23].

We attributed the OAI ICG signal to the dye as a specific peak at 800 nm could be observed and excretion of the dye was confirmed by fluorescence imaging. Therefore, the discrepancy between both cohorts might also result from the ETI treatment. However, spectral unmixing of ICG could also be influenced by dosing and the in vivo environment [35]. Despite the observed positive effect of ETI treatment, gastrointestinal symptoms still prevail in the majority of pwCF in the CFTR modulator era [29]. Thus, there is a critical need for non-invasive and radiation-free modalities for examining the gastrointestinal tract. As MSOT provides a dynamic visualization of the passage of bowel contents, it has the potential to become a valuable imaging technique for diagnosing and monitoring gastrointestinal aspects in pwCF [23]. The findings of this work suggest its implementation into everyday clinical practice of pwCF, potentially reducing the need for more invasive procedures. To refine this method and establish a clinically applicable standard, follow-up studies encompassing a larger cohort of pwCF, including pediatric populations, are necessary.

This study has several limitations to consider. It is constrained by a small and heterogeneous sample size. So our results can be seen as a first investigation in this field, but should be subject to confirmation in more extensive trials. Due to the small sample size, it is crucial to interpret the results cautiously, considering the potential for significant variability in p-values in small cohorts. Moreover, MSOT has currently a maximum penetration depth of approximately 2.5 cm, limiting its imaging capabilities of deep-lying organs, particularly in patients with high body mass indices (BMI). Additionally, gender-specific variations in natural fat distribution can pose a challenge in clinical application [36]**.**

Nevertheless, it holds great potential to become an important part of clinical care, especially in pediatric patients where radiation-free tools are warranted. Our findings demonstrate that CE-MSOT could be used a useful screening and diagnostic tool in CF patient care. As optoacoustic imaging systems are still a novel imaging approach, they need to be further improved and standardized [37]. Further research is required to refine MSOT techniques, standardize examination protocols, and investigate its broader applicability in the clinical management of CF.

## Author contributions

AS and APR designed the study. AS, JF, APR and FW performed the ultrasound and optoacoustic tomography examinations. JF, FW, AS and APR analyzed the data. JF and MC performed statistical analyses. JF, AH, FK, AS and APR interpreted the data. JF wrote the first draft of the manuscript. FW, MC, HV, LPP, HM, AB, GS, JJ, JW, AH, FK, AS and APR reviewed the manuscript. All authors approved the final version.

## CRediT authorship contribution statement

**Hannah Vogt-Wolz:** Writing – review & editing, Visualization, Project administration. **Lars-Philip Paulus:** Writing – review & editing, Methodology. **Henriette Mandelbaum:** Writing – review & editing, Supervision, Project administration. **Adrian Buehler:** Writing – review & editing, Visualization, Methodology. **André Hörning:** Writing – review & editing, Supervision. **Johanna Fuchte:** Writing – original draft, Investigation, Formal analysis. **Ferdinand Knieling:** Writing – review & editing, Supervision, Resources, Project administration, Conceptualization. **Felix Wachter:** Writing – review & editing, Supervision, Project administration, Investigation, Conceptualization. **Adrian P Regensburger:** Writing – original draft, Supervision, Software, Resources, Project administration, Methodology, Investigation, Funding acquisition, Formal analysis, Data curation, Conceptualization. **Merle Claßen:** Writing – review & editing, Software, Formal analysis, Data curation. **Alexander Schnell:** Writing – original draft, Supervision, Project administration, Investigation, Funding acquisition, Formal analysis, Data curation, Conceptualization. **Gregor Siebenlist:** Writing – review & editing, Investigation. **Jörg Jüngert:** Writing – review & editing, Supervision, Investigation. **Joachim Wölfle:** Writing – review & editing, Supervision.

## Funding

The Else Kröner-Fresenius Stiftung partially funded this project to APR (Excellence Fellowship), the Interdisciplinary Center for Clinical Research (IZKF) at the University Hospital of the Friedrich-Alexander-Universität (FAU) partially funded this project to AS (J108 and CSP program), and the 10.13039/501100000780European Union to FK (ERC starting grant No. 101115742).

## Declaration of Competing Interest

The authors declare that they have no known competing financial interests or personal relationships that could have appeared to influence the work reported in this paper.

## Data Availability

Data will be made available on request.
