# Supplementary material for: Contrast-enhanced multispectral optoacoustic tomography for the assessment of the gastrointestinal transit in patients with cystic fibrosis
Source: Photoacoustics. 2025 Sep 10;46:100766. doi: 10.1016/j.pacs.2025.100766 (PMC12745951; doi:10.1016/j.pacs.2025.100766)
Supplement: Supplementary file 1 — Supplementary material [file mmc1.docx]

Supplementary Appendix

The present supplementary appendix should give additional information to the readers of

**“Contrast-enhanced Multispectral optoacoustic tomography for the assessment of the gastrointestinal transit in patients with cystic fibrosis”** by

Johanna Fuchte^1^, Felix Wachter^1^, Merle Claßen^1^, Hannah Vogt^1^, Lars-Philip Paulus^1^, Henriette Mandelbaum^1^, Adrian Buehler^1^, Gregor Siebenlist^1^, Jörg Jüngert^1^, Joachim Wölfle^1^, André Hoerning^1^, Ferdinand Knieling^1^, Adrian P Regensburger^1^*, Alexander Schnell^1^*

^1^ Department of Pediatrics and Adolescent Medicine, University Hospital Erlangen, Germany

**TABLE OF CONTENTS**

[Supplementary Tables 4](#_Toc206528852)

[Supplementary Table 1 – Exclusion criteria of screened CF patients 4](#_Toc206528853)

[Supplementary Table 2 – CFabd- Score 5](#_Toc206528854)

[Supplementary Figures 6](#_Toc206528855)

[Supplementary Figure 1 – MSOT ICG signal quantification in the gastric antrum 6](#_Toc206528856)

[Supplementary Figure 2 – Spectrum of MSOT ICG signal quantification 7](#_Toc206528857)

[Supplementary Figure 3 – Spectrum of Stool and ICG signal quantification 8](#_Toc206528858)

[Supplementary Figure 4 – MSOT ICG signal quantification of single study participants in the terminal ileum 9](#_Toc206528859)

[Supplementary Figure 5 – Absolute values of MSOT ICG signal quantification in the terminal ileum 10](#_Toc206528860)

[Supplementary Figure 6 – MSOT ICG signal quantification of single study participants in the sigmoid colon 11](#_Toc206528861)

[Supplementary Figure 7 – Absolute values of MSOT ICG signal quantification in the sigmoid colon 12](#_Toc206528862)

# Supplementary Tables

## Supplementary Table 1 – Exclusion criteria of screened CF patients

| **Excluded Patients** | **Reason of exclusion** |
| --- | --- |
| 1 | no interest in participation (personal reasons) |
| 2 | no interest in participation (personal reasons) |
| 3 | only baseline measurements were carried out |
| 4 | not of legal age |
| 5 | no interest in participation (personal reasons) |
| 6 | no interest in participation |
| 7 | abroad at the time of the study |
| 8 | preparing for a liver transplant |
| 9 | no interest in participation (personal reasons) |
| 10 | no interest in participation (personal reasons) |
| 11 | no interest in participation (personal reasons) |
| 12 | no time for participation (personal reasons) |
| 13 | no time for participation (personal reasons) |
| 14 | no time for participation (personal reasons) |
| 15 | no time for participation (personal reasons) |
| 16 | no interest in participation (personal reasons) |

**Supplementary Table 1 – Exclusion criteria of screened CF patients**

Various reasons led to the exclusion of the screened patients, however 16 of 22 screened patients were excluded.

## Supplementary Table 2 – CFabd- Score

|  | HC | CF |  |
| --- | --- | --- | --- |
| Abdominal pain frequency | 1.0 ± 1.4 | 0.8 ± 0.4 | (p = 0.76) |
| Abdominal pain intensity | 1.0 ± 1.4 | 2.6 ± 2.6 | (p = 0.40) |
| Flatulence frequency | 1.8 ± 1.0 | 2.6 ± 1.1 | (p = 0.38) |
| Obstipation frequency | 1.3 ± 1.5 | 0.8 ± 0.8 | (p = 0.80) |
| Steatorrhea | 0 ± 0 | 2.0 ± 2.7 | (p = 0.44) |
| Defecation frequency | 0 ± 0 | 0.8 ± 0. 4 | (**p* < 0.05) |
| Defecation consistency | 0 ± 0 | 1.8 ± 1.6 | (p = 0.17) |
| Defecation colour | 0 ± 0 | 1.0 ± 2.2 | (p > 0.99) |
| Total score | 5.4 ± 3.8 | 11.4 ± 5.5 | (p = 0.13) |

**Supplementary Table 2 – CFabd- Score**

CFabd- Score items were scaled as follows: Abdominal pain frequency: 0 never, 1 very rare, 2 rare, 3 occasionally, 4 frequently, 5 daily; Abdominal pain intensity: 0 no pain, 10 worst pain imaginable; Steatorrhea: 0 yes, 5 no; Defecation frequency: 1x/week (2) - 2x/week (1), every second day (0) - 1x daily (0), 2x/day (1) - >2x/day (2); Defecation consistency: 0 shaped, 1 hard, 3 hard and mushy, 5 mushy and liquid; Defecation colour: 1 normal coloured, 3 dark (black and tarry), 5 pale and discoloured.

# Supplementary Figures

## **Supplementary Figure 1 – MSOT ICG signal quantification in the gastric antrum**


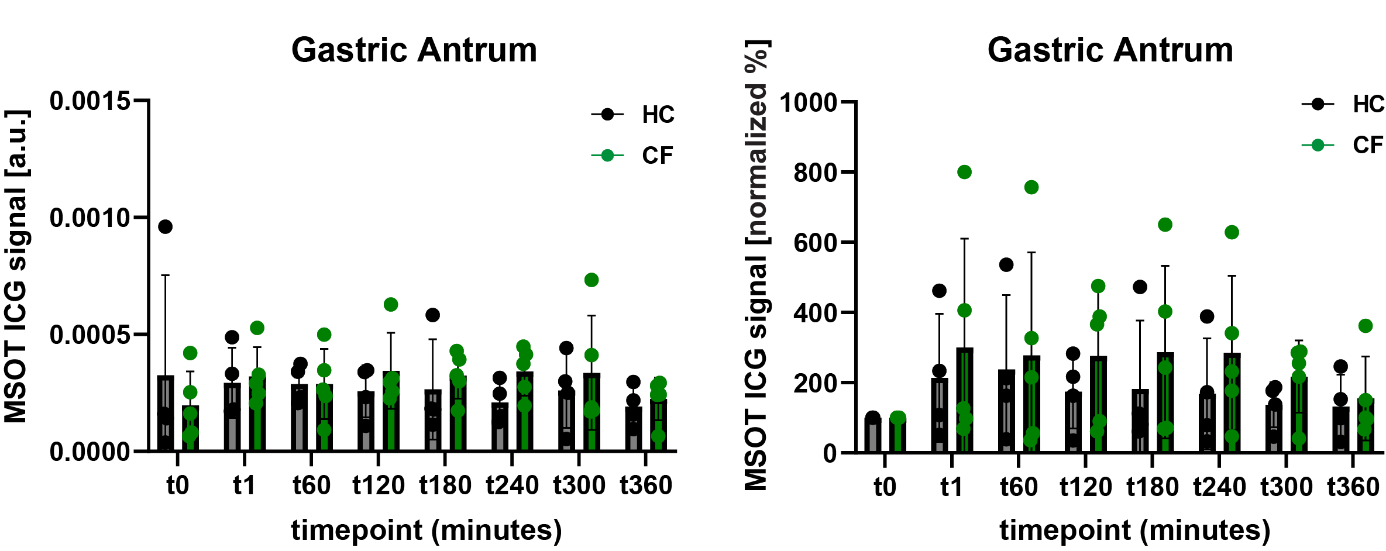


**Supplementary Figure 1 - MSOT ICG signal quantification in the gastric antrum**

Quantification of the MSOT- ICG signal of each imaging timepoint of the day in the gastric antrum, one in healthy subjects (black) and one in people with Cystic Fibrosis (green). Dots represent single values of each study participant, bars and whiskers represent mean ± SD. HC = Healthy Controls, CF = Cystic Fibrosis.

## Supplementary Figure 2 – Spectrum of MSOT ICG signal quantification


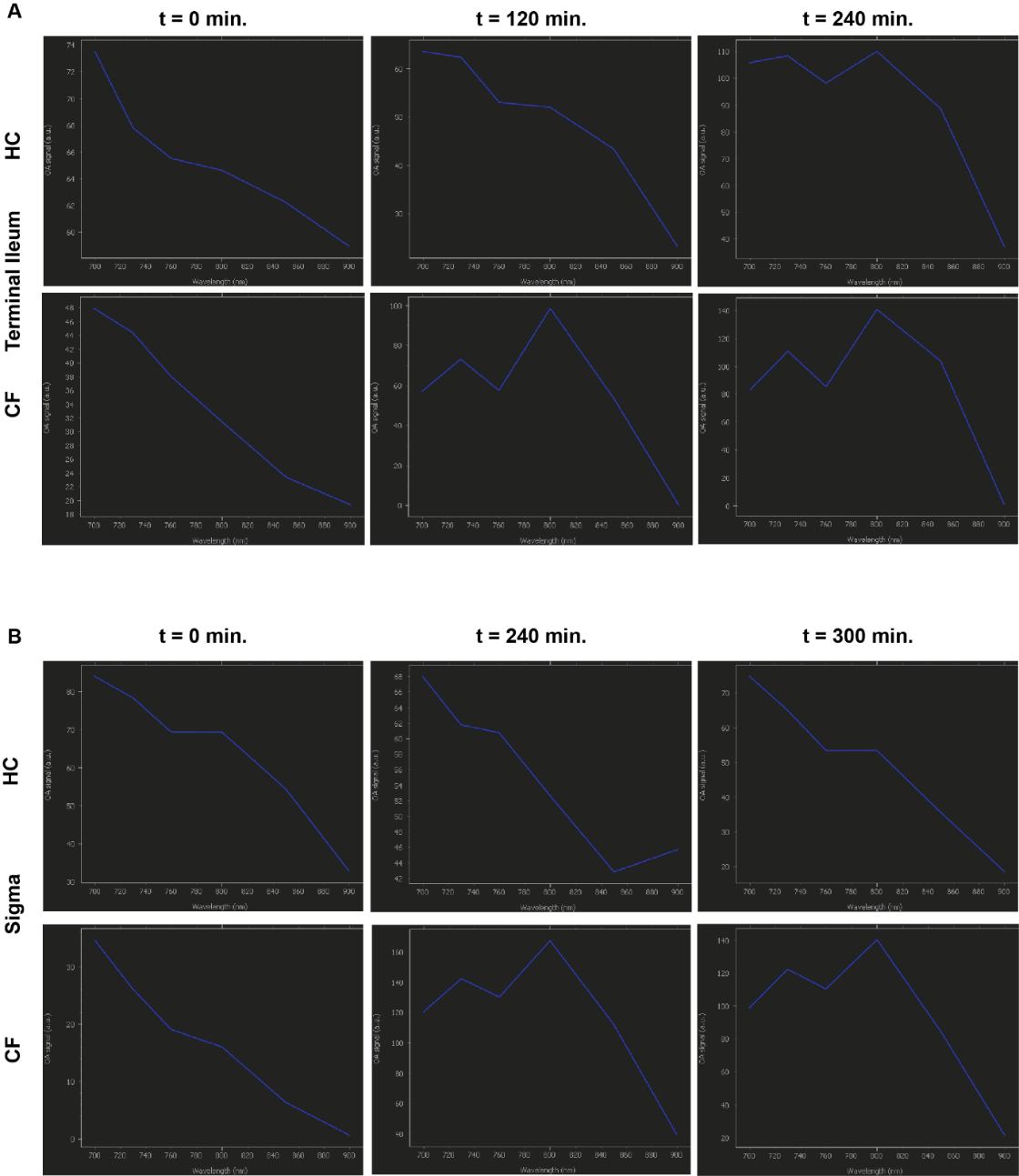


Supplementary Figure 2 – Spectrum of MSOT ICG signal quantification

To differentiate ICG signals from background signals, the absorption spectrum within the regions of interest from the displayed images in Figure 3 were compared. HC = Healthy Controls, CF = Cystic Fibrosis patients.

A) Terminal Ileum: Measurements in which an OAI ICG signal was not suspected (t= 0 and t=120 min in HC and t= 0 in pwCF) exhibited varying spectral profiles. In contrast, the optoacoustic spectra obtained when a true ICG signal was suspected closely matched the spectral profile of ICG itself (t= 120 and t = 240 min in pwCF, t= 240 min in HC).

B) Sigma: Measurements in which an OAI ICG signal was not suspected (e.g., t= 0 in HC and CF, t= 240 and t= 300 in HC) exhibited varying spectral profiles. In contrast, the optoacoustic spectra obtained when a true ICG signal was suspected closely resembled the spectral profile of ICG itself (t= 240 and t= 300 in CF).

## Supplementary Figure 3 – Spectrum of Stool and ICG signal quantification


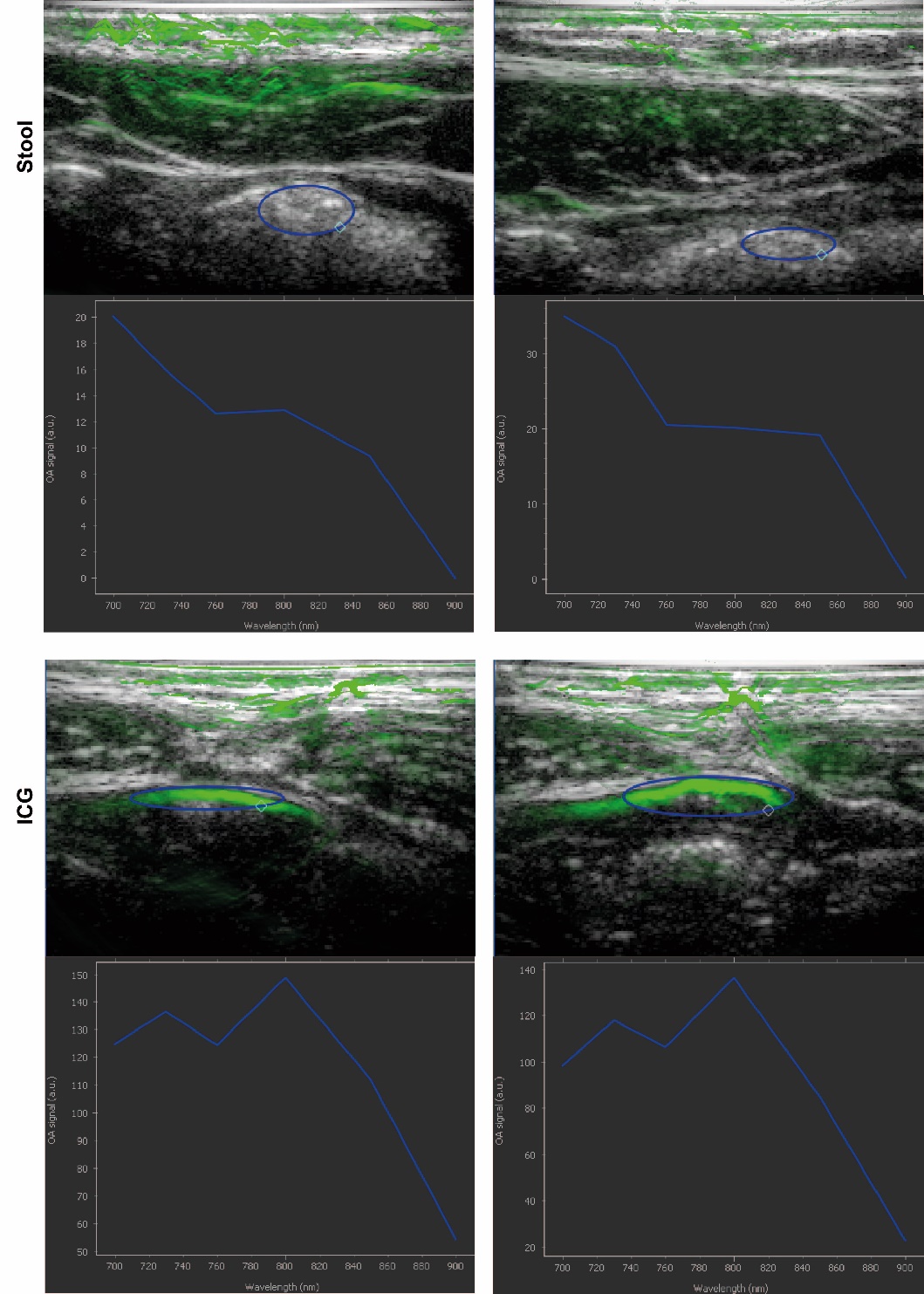


Supplementary Figure 3 – Spectrum of Stool and ICG signal quantification

In order to clearly distinguish ICG signals from intrinsic stool signals, we analyzed the respective spectra. While ICG exhibits a characteristic peak at 800nm, this peak is not detectable in stool. Supplementary Figure 3 shows MSOT images of stool without ICG (top) and stool with ICG (bottom) signal within the region of interest (blue ellipse). The corresponding spectrum is displayed below the MSOT image. There is a clear difference between the spectrum of the stool with and without ICG.

## Supplementary Figure 4 – MSOT ICG signal quantification of single study participants in the terminal ileum


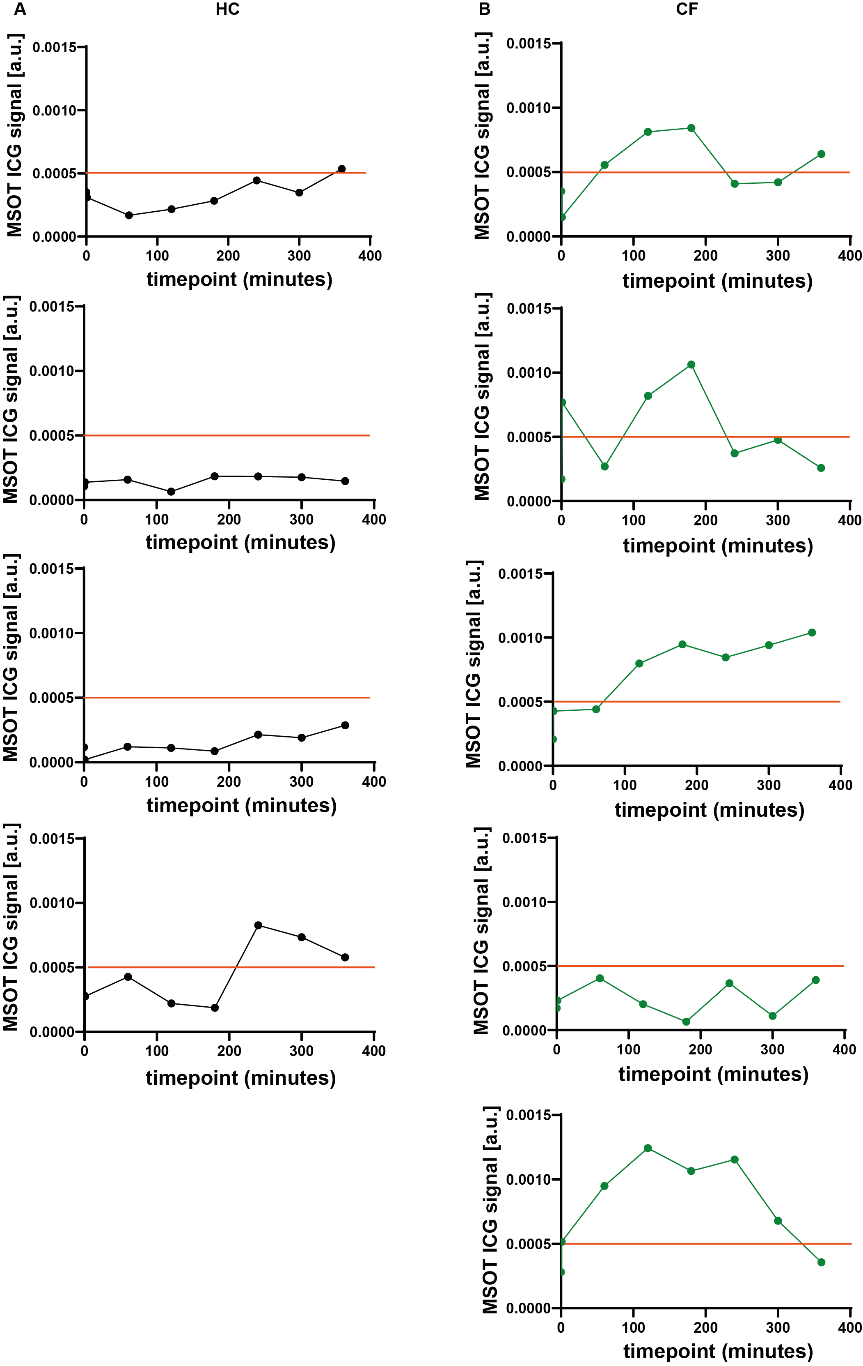


Supplementary Figure 4 – MSOT ICG signal quantification of single study participants in the terminal ileum

Supplementary Figure 4 shows the ICG curves for each individual study participant in the terminal ileum (left HC and right CF). The highest measured baseline ICG value at timepoint 0 (false positive) was used as cut off (horizontal orange line). Values exceeding this cut off were attributed to the dye ICG. HC = Healthy Controls, CF = Cystic Fibrosis.

## Supplementary Figure 5 – Absolute values of MSOT ICG signal quantification in the terminal ileum


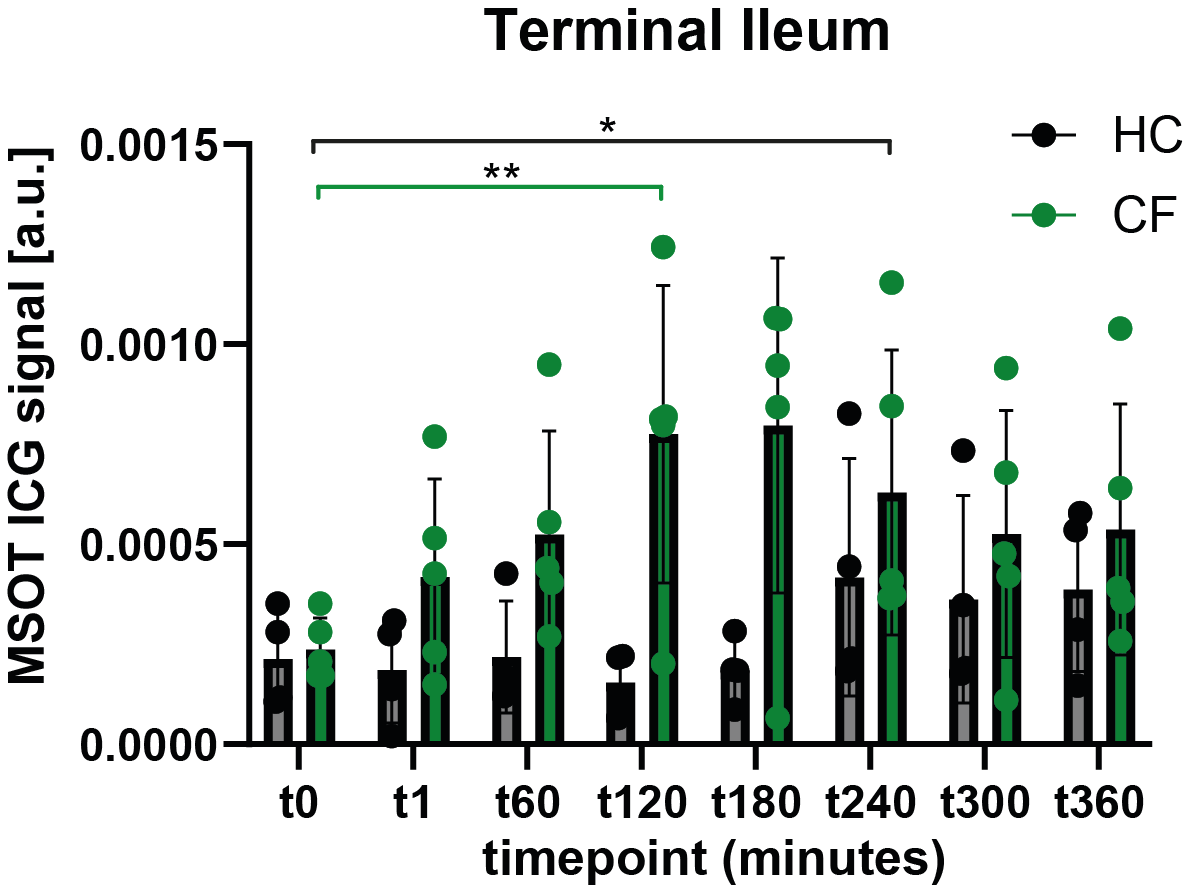


**Supplementary Figure 5 – Absolute values of MSOT ICG signal quantification in the terminal ileum**

Quantification of the MSOT-ICG signal of each imaging timepoint of the day in the terminal ileum, one in healthy subjects (black) and one in people with Cystic Fibrosis (green). Dots represent single values of each study participant, bars and whiskers represent mean ± SD. HC = Healthy Controls, CF = Cystic Fibrosis.

## Supplementary Figure 6 – MSOT ICG signal quantification of single study participants in the sigmoid colon


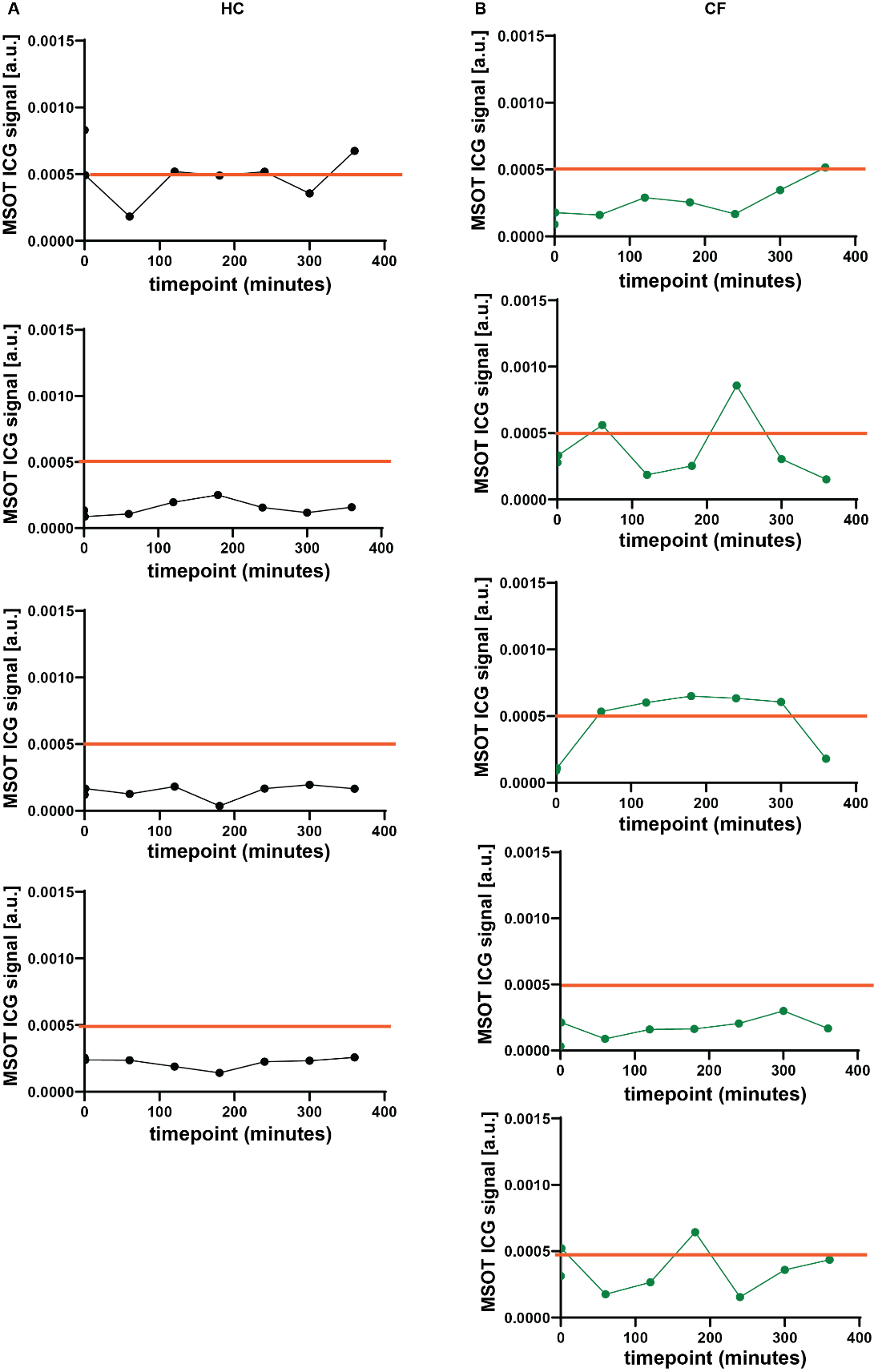


Supplementary Figure 6 – MSOT ICG signal quantification of single study participants in the sigmoid colon

Supplementary Figure 6 shows the ICG curves for each individual study participant in the sigmoid colon (left HC and right CF). The OAI ICG cut off is marked with a horizontal orange line. Values exceeding this cut off were attributed to the dye ICG. HC = Healthy Controls, CF = Cystic Fibrosis.

## Supplementary Figure 7 – Absolute values of MSOT ICG signal quantification in the sigmoid colon


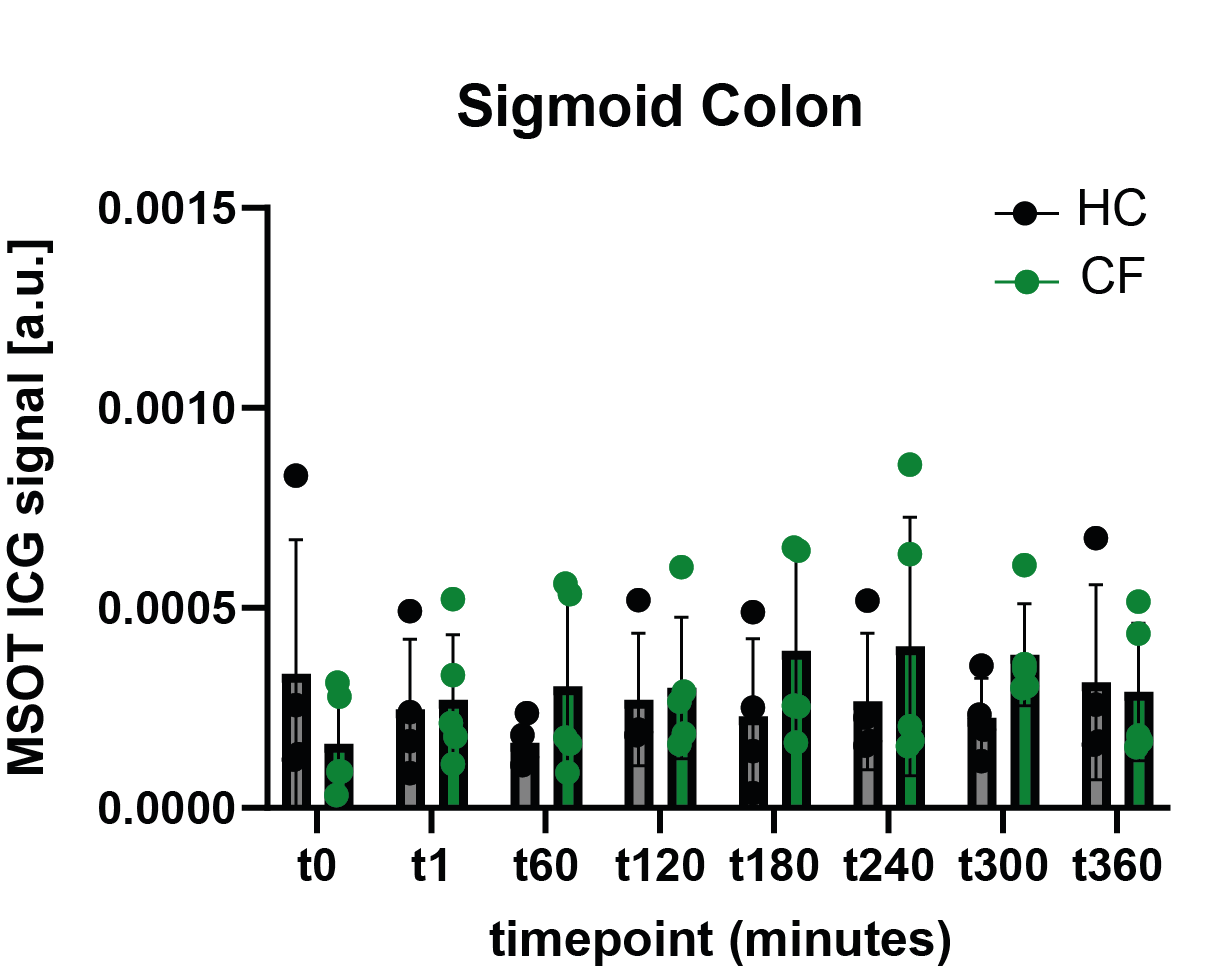


**Supplementary Figure 7 – Absolute values of MSOT ICG signal quantification in the sigmoid colon.**

Quantification of the MSOT-ICG signal of each imaging timepoint of the day in the sigmoid colon, one in healthy subjects (black) and one in people with Cystic Fibrosis (green). Dots represent single values of each study participant, bars and whiskers represent mean ± SD. HC = Healthy Controls, CF = Cystic Fibrosis.
